# A Tunable Coarse-Grained Model for Ligand-Receptor Interaction

**DOI:** 10.1371/journal.pcbi.1003274

**Published:** 2013-11-14

**Authors:** Teresa Ruiz-Herrero, Javier Estrada, Raúl Guantes, David G. Miguez

**Affiliations:** 1 Departamento de Física Teórica de la Materia Condensada, Universidad Autónoma de Madrid, Madrid, España; 2 Departamento de Física de la Materia Condensada, Instituto de Ciencias de Materiales Nicolás Cabrera, Universidad Autónoma de Madrid, Madrid, España; 3 Condensed Matter Physics Center (IFIMAC), Universidad Autónoma de Madrid, Madrid, España; University of Maryland, Baltimore, United States of America

## Abstract

Cell-surface receptors are the most common target for therapeutic drugs. The design and optimization of next generation synthetic drugs require a detailed understanding of the interaction with their corresponding receptors. Mathematical approximations to study ligand-receptor systems based on reaction kinetics strongly simplify the spatial constraints of the interaction, while full atomistic ligand-receptor models do not allow for a statistical many-particle analysis, due to their high computational requirements. Here we present a generic coarse-grained model for ligand-receptor systems that accounts for the essential spatial characteristics of the interaction, while allowing statistical analysis. The model captures the main features of ligand-receptor kinetics, such as diffusion dependence of affinity and dissociation rates. Our model is used to characterize chimeric compounds, designed to take advantage of the receptor over-expression phenotype of certain diseases to selectively target unhealthy cells. Molecular dynamics simulations of chimeric ligands are used to study how selectivity can be optimized based on receptor abundance, ligand-receptor affinity and length of the linker between both ligand subunits. Overall, this coarse-grained model is a useful approximation in the study of systems with complex ligand-receptor interactions or spatial constraints.

## Author Summary

The current importance of cell surface receptors as primary targets for drug treatment explains the increasing interest in a mathematical and quantitative description of the process of ligand-receptor interaction. Recently, a new generation of synthetic chimeric ligands has been developed to selectively target unhealthy cells, without harming healthy tissue. To understand these and other types of complex ligand-receptor systems, conventional chemical interaction models often rely on simplifications and assumptions about the spatial characteristics of the system, while full atomistic molecular dynamics simulations are too computationally demanding to perform many particle statistical analysis. In this paper, we describe a novel approach to model the interaction between ligands and receptors based on a coarse grained approximation that includes explicitly both spatial and kinetic details of the interaction, while allowing many-particle simulations and therefore, statistical analysis at biologically relevant time scales. The model is used to study the binding properties of generic chimeric ligands to understand how cell specificity is achieved, its dependence on receptor concentration and the influence of the distance between subunits of the chimera. Overall, this approach proves optimal to study other ligand-receptor systems with complex spatial regulation, such as receptor clustering, multimerization and multivalent asymmetric ligand binding.

This is a *PLOS Computational Biology* Methods article.

## Introduction

Extracellular signals, such as morphogens and hormones, bind to specific receptors on the cell surface to activate signaling cascades that ultimately regulate key cell decisions, such as proliferation, migration or apoptosis. In multicellular organisms, dysregulation of this receptor-initiated signaling can lead to uncontrolled cell proliferation and cancer. Nowadays, around 60% of all commercial drugs are designed to target specific receptors on the cell surface. Due to this role in stimulus recognition and upstream regulation of cell signaling, mathematical modeling of ligand-receptor interaction constitutes a major effort in the development and rational design of novel therapeutic strategies. The majority of these models are based on a chemical kinetics description of ligands in a three-dimensional environment that bind to receptors diffusing in a two-dimensional surface [1], [2]. In more complex scenarios [3], [4] where the spatial constraints of the interaction are important, the reaction rates are assumed to be simply modulated by receptor diffusion, while interactions at the membrane level are assumed to be facilitated by the reduction in the dimensionality of the system, following Adam and Delbruck seminal contribution [5].

Although these assumptions may be valid in simple scenarios, they neglect several important regulatory mechanisms induced by the structural details of the interaction, such as diffusion inhomogeneities or conformational changes after multimerization, or sequential binding [3]. Alternative computational approaches use a detailed atomistic description of both ligands and receptors [6]–[9] to fully account for the spatial regulation in the interaction, such as receptor orientation or ligand asymmetry. Unfortunately, such models require large computational resources, making prohibitive the implementation of many-particle simulations for statistical analysis or the implementation of slow degrees of freedom, such as receptor diffusion. Other more computationally efficient approaches reduce some degrees of freedom using hybrid models where some parts of the system, usually the membrane or regions of the receptor, are coarse-grained [10], [11]; applying Monte Carlo simulations with statistical reconstructions to reproduce the dynamics of the ligand-receptor interaction [12]; or taking advantage of lattice models where both binding/unbinding events follow a given probability [13]–[15].

In this work we present a coarse-grained approximation to ligand-receptor interactions, which allows for a computationally feasible study of the dynamics of systems with different sets of ligands and receptors at biologically relevant temporal and spatial scales. The model takes into account both spatial and kinetic features of the interaction, while allowing many particle simulations, and statistical analysis. The interaction between ligand and receptor is described by two basic parameters (angular specificity and strength) which can be tuned to fit a broad range of affinities and dissociation rates to model different ligand-receptor pairs. First, we show that the kinetics of ligand binding and unbinding behave as predicted by chemical kinetics theory, in terms of diffusion and receptor abundance. Then we correlate the parameters defining the interaction (angular specificity and strength) with the experimentally relevant affinity and dissociation rate parameters. As a case study, the model is applied to the analysis of the binding properties of generic chimeric ligands, where we show how cell specificity is achieved and how it depends on receptor diffusion and length of the linker between ligand subunits of the chimera.

## Methods

Using a coarse-grained approximation, the ligand-receptor interaction is characterized by the geometry of the reactants and by the chemical nature of their binding. The first feature sets the interaction specificity by inducing a steric contribution that limits the binding region to a specific area of the receptor molecule; the chemical contribution, in turn, sets the strength of the bond.

Within this representation, ligands and receptors are simplified as spherical particles of diameters 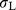 and 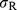 respectively. Particles of the same type interact via a repulsive WCA potential [16]:
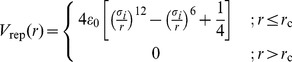
(1)with 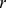 being the distance between particles, 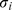 the diameter of the reactant type 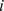 (either 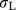 or 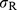), and 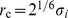 the cutoff distance for the potential. For the simulations, we set 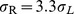 to mimic the typical size ratio between epidermal growth factor ligands and its complementary receptor [17]. Results are shown in terms of reduced units, where 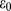 and 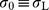 are the characteristic energy and length units of the system, respectively.

To account for the interaction between ligand and receptor 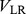, we use an angular-dependent form of a generic Lennard-Jones potential which takes non-zero values for 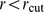


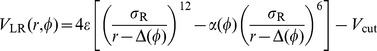
(2)where 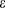 is the interaction strength, 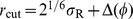, and 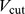 is set such that the interaction is zero at the cutoff, 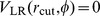.

The functions 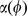 and 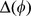 are defined as follows:
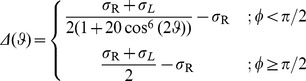
(3)

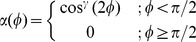
(4)


These functions are chosen so that they and their derivatives are continuous at 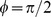 and represent a smooth angular dependency with 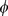. For 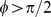, 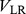 is reduced to the repulsive term of the Lennard-Jones. The parameter 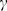 can be understood as a geometric factor that modulates the angular specificity of the interaction.

**Figure 1:**
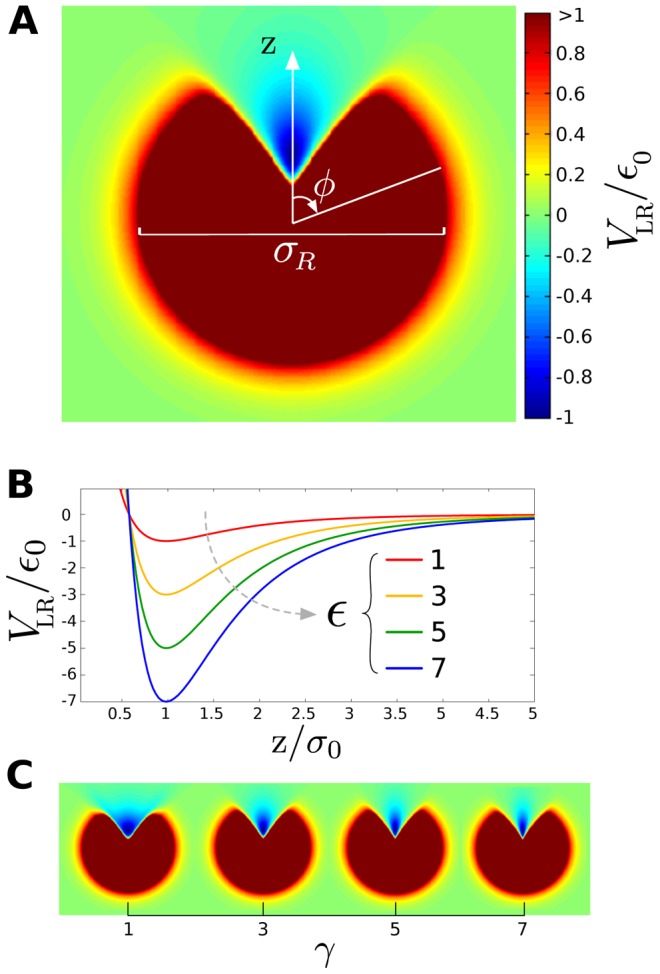
Spatial characteristics of the ligand-receptor interaction. **A**) Profile of the ligand-receptor potential 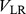 for 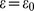 and 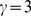. Attractive interaction is plotted in blue, while repulsive interaction is represented in yellow and red. **B**) Effect of the interaction parameter 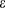 on the interaction strength along the z axis. The binding energy increases with 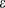. **C**) Effect of the geometric coefficient 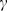 on the ligand-receptor interaction. The binding area decreases with 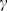.

The ligand-receptor interaction potential can be identified as the effective shape of the receptor seen by a ligand molecule (Fig. 1A). The effect of the parameters 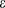 and 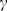 on the interaction strength and the binding area are showed in Fig. 1B and Fig. 1C.

The ligand-receptor interaction is studied using molecular dynamics simulations where ligands of reduced mass 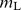 follow Langevin dynamics with diffusion coefficient 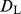 at constant temperature given by 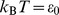 [18]. These magnitudes fix the characteristic time-scale of the system 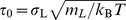. Receptor diffusion within the membrane is much slower than ligand diffusion, so it is neglected during the study of the monovalent binding of ligands to receptors, but will be taken into account in the characterization of chimeric ligands (see *Results*). Receptor diffusion is implemented following the Langevin formalism, using a receptor mass 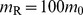 and diffusion coefficient 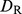. For the initial configuration, the number of receptors 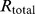 is fixed (typically between 100–200) and they are randomly distributed in the x-y plane of a box with dimensions 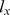, 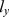 and 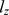. The lateral dimensions of the box are calculated, given a receptor concentration per unit area 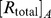, as 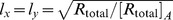. The magnitude of 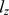 is adjusted to facilitate analysis depending on the process studied (see *Results*). In order to avoid finite size effects, periodic boundary conditions are applied.

Typically, 4 to 32 independent simulations are run for each set of parameters, and the results are averaged to calculate rate constants. Standard deviations due to system fluctuations are represented by error bars. Results are plotted in terms of the characteristic length 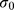, energy 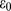, and time 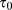 of the system. Volume concentrations are denoted by variables in brackets, while the sub-index “
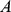
” indicates surface concentrations. Sub-index “
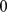
” corresponds to initial values.

Simulations were performed using in-house code where the model was implemented in a Verlet algorithm (source code available as Supplementary Material Software S1 in this manuscript). Given the simplicity of the model, simulations were ran in a regular desktop computer. For a system with 300 receptors and 500 chimeras (1000 ligands), one million steps were performed in 20 min using one core at 2.76 GHz, this is a performance of around 830 TPS (time steps per second).

## Results

### Characterization of simple ligand-receptor interactions

To validate our coarse-grained model, we first apply it to characterize simple ligand-receptor interactions. The chemical interaction between a freely diffusing ligand 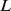 and its corresponding receptor 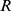 that lies on the cell membrane to form a complex 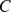 follows the reaction scheme:
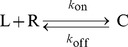
(5)where 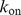 and 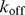 correspond to the affinity and dissociation rates. Using mass-action kinetics, the dynamics of the system are described by a simple ordinary differential equation:
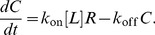
(6)


**Figure 2:**
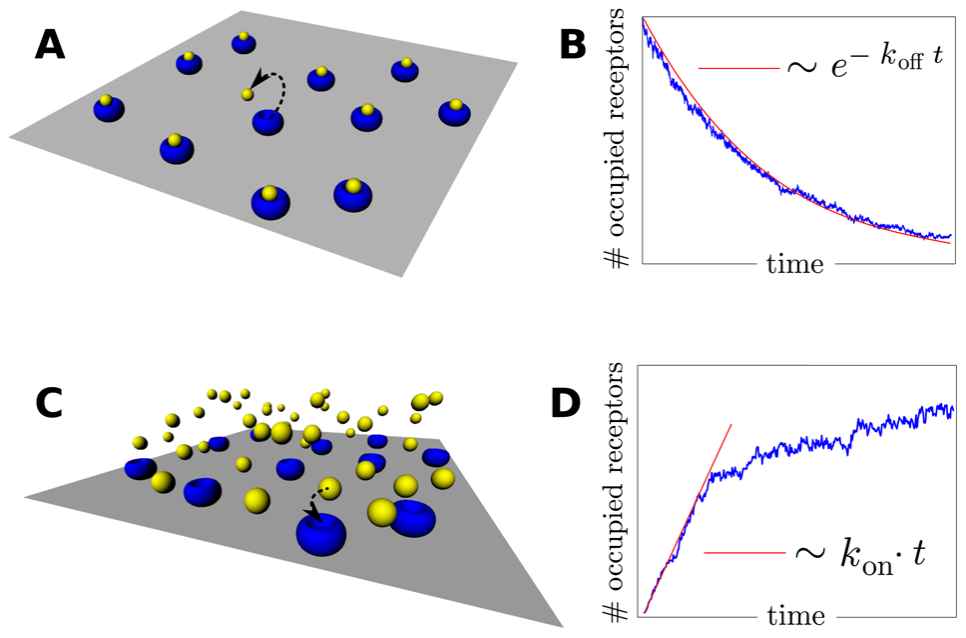
Affinity and dissociation simulations. **A**) Schematic of the initial configuration of ligands and receptors to calculate the dissociation rate. **B**) Dynamics of ligand-receptor dissociation follows a exponential decay that allows to calculate the dissociation constant 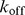. **C**) Schematic of the initial configuration of ligands and receptors to calculate the affinity rate. **D**) Dynamics of ligand-receptor affinity follows a linear growth at short times following Eq. (8).

One of the main advantages of our modeling framework is that both rate constants can be directly computed using statistical many-particle analysis: for the dissociation rate constant 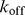, molecular dynamics simulations are performed with initially all receptors bound (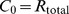) and no free ligands (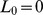). These complexes (200 per simulation run) were placed in a box with a large 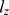 size (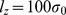) and receptor density 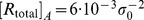 to avoid rebinding events (Fig. 2A). In this situation, the first term in Eq.(6) is zero and the average amount of complexes decays as:
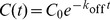
(7)


We thus fit the amount of complexes as a function of time to an exponential to estimate 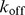 values (Fig. 2B).

To calculate 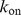, we initially set all available receptors unbound, and then monitor the formation of complexes as a function of time (Fig. 2C). The simulations were done with 100 receptors at high ligand concentration, 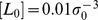, 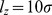, and 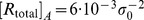. The affinity rate at steady state can be calculated from the equilibrium condition in Eq. (6). However, at short times (before dissociation events start to be relevant) the solution of Eq. (6) with 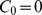 can be approximated as
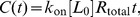
(8)so 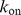 can be directly obtained from a linear fit in this regime (Fig. 2D). We checked that both long and short time estimates of 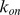 produced the same results within numerical accuracy.

### Dependence of affinity and dissociation rates on the simulation parameters

**Figure 3:**
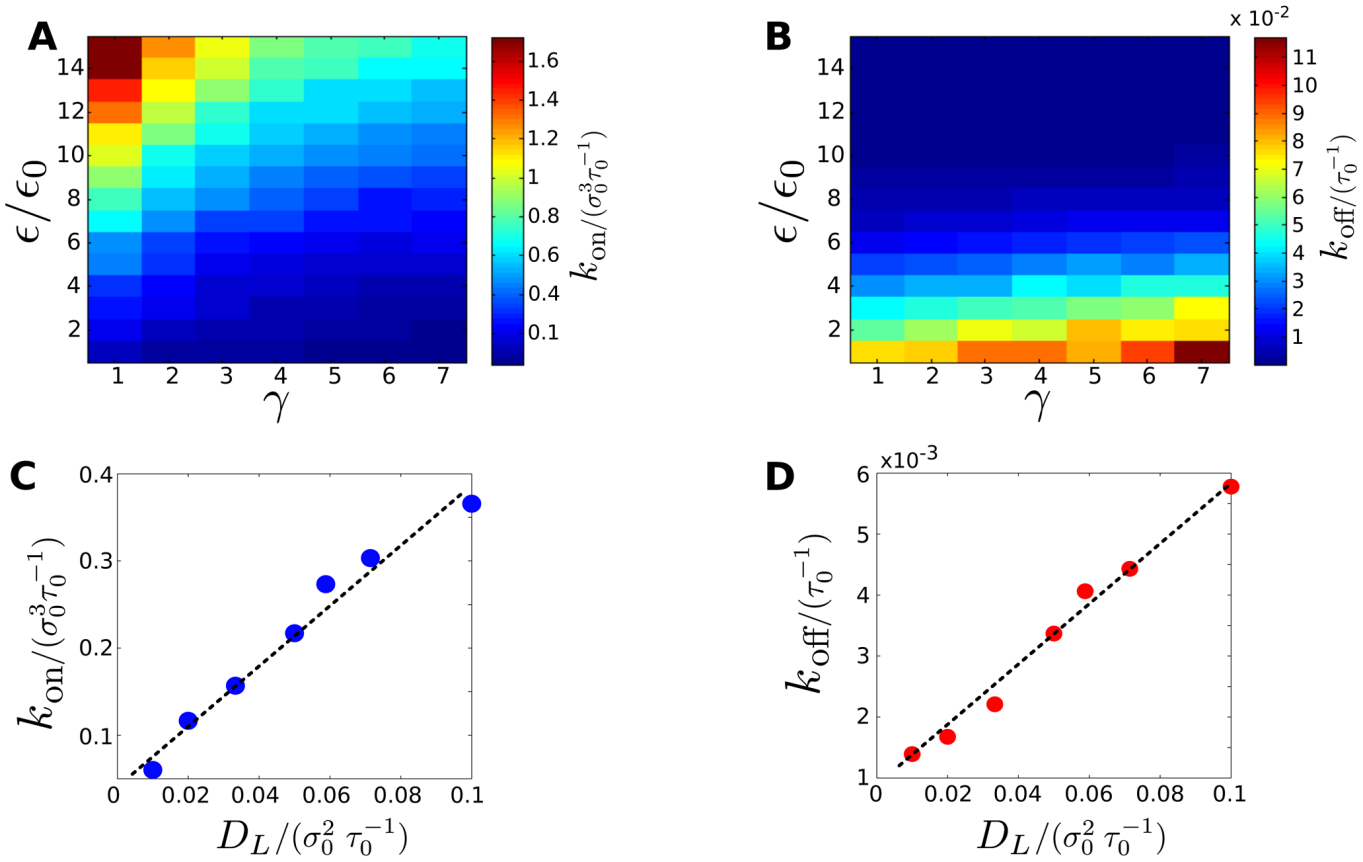
Dependence of the reaction rates on the system parameters. (**A** and **B**): Dependence of 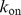 and 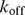 with the tuning parameters for 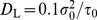 and 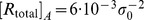. 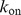 (**A**) was determined via association experiments with 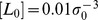 and 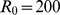. 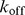 (**B**) was determined via dissociation experiments with 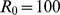. **C** and **D**): 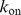 and 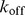 as a function of the diffusion coefficient 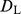 for 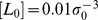, 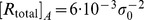, 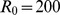, 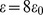 and 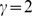. Dashed lines: linear fit for the dependence of the reaction rates with the diffusion coefficient 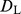, with slopes: 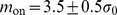, 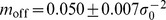. In all four panels each outcome is the result of 4 independent simulations and has an error 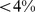. In panels C and D, error bars are within the size of the symbols.

To investigate how the affinity/dissociation rates depend on the two main parameters of our model, we calculate 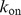 and 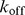 varying simultaneously 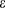 (interaction strength factor) and 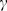 (geometric factor) in a significant range. The affinity rate 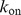 shows a strong dependence on both parameters (Fig. 3A), since binding depends on the depth of the Lennard-Jones potential and the available attractive area on the receptor surface. On the contrary, 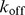 shows a weak dependence on the geometric factor 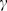 (Fig. 3B) since, after the ligand is bound to the receptor, dissociation depends mainly on the depth of the attractive well through a Boltzmann factor, while geometrical aspects such as the curvature of the dissociation barrier [19] play a minor role. Due to this weak dependence of 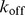 on 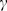, both free parameters 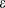 and 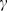 can be tuned to fit different combinations of 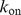 and 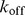; therefore our model can be easily tailored to represent different ligand-receptor systems.

Other aspects of the monovalent ligand-receptor system can be characterized within our modeling framework, such as the effect of ligand diffusion on the binding kinetics. The measured kinetic rates 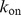 and 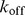 depend on diffusion through a transport rate constant 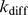 as [20], [21]:
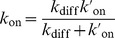
(9)

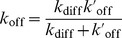
(10)


Here, 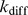 is proportional to the diffusion constant of the ligand 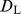 [22], [23], while 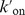 and 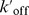 state for the intrinsic reaction rates. We obtained that both affinity and dissociation rates exhibit a linear dependency with the diffusion coefficient (Fig. 3C–D). This indicates that, for the parameters used, the system is operating in a diffusion-limited regime (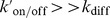) and therefore 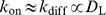. This behavior is consistent with an scenario where typical affinity/dissociation time-scales are much faster than diffusion time-scales.

Overall, we have shown that the coarse-grained model reproduces the main aspects of the ligand-receptor interaction, and provides a good description to statistically obtain the affinity and dissociation rates via dynamical simulations. We next apply our model to characterize more complex ligand-receptor scenarios.

### Coarse-grained model to study the dynamics of chimeric ligands

**Figure 4:**
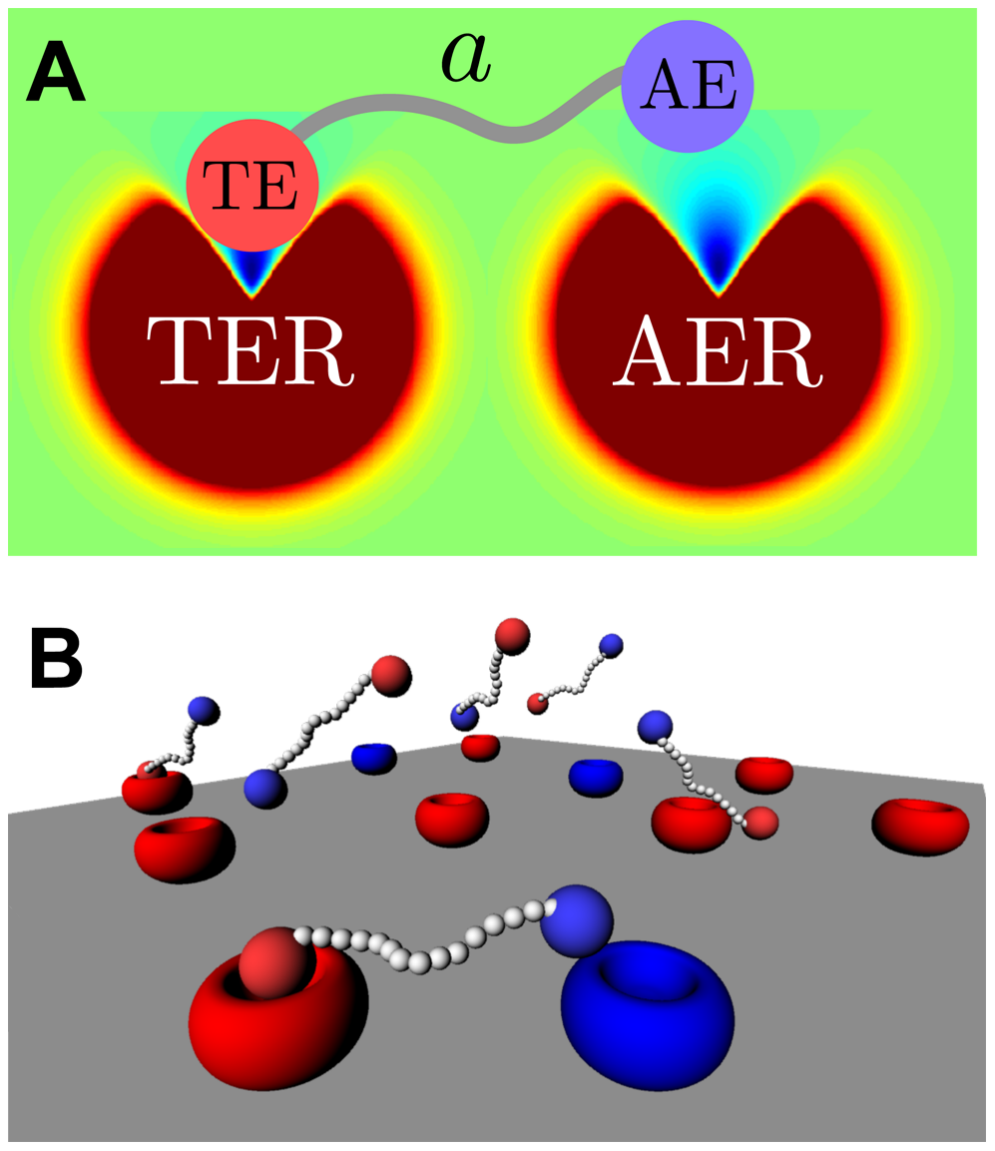
Schematic of chimeric binding. **A**) Binding of activity elements (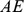
's) to activity element receptors (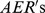) is mediated by the binding of the target elements (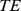
's and 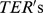) via a polymer chain of length 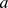. **B**) Schematic of a multi component system with receptors diffusing within a surface, and freely diffusing chimeras. The colors represent different species, while the polymer chain is approximated via a worm-like chain potential.

One of the most promising strategies to improve the efficiency and selectivity of drug-based therapies is the use of synthetic chimeric ligands [24]–[26]. Typically, these chimeras consist of two subunits: an activity element (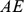) that triggers a desired cellular response, by interacting with a specific receptor (for instance a receptor involved in an apoptotic signaling cascade), and a targeting element (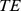) that binds to a receptor differentially expressed in healthy *versus* unhealthy cells, (Fig. 4). The efficiency of these chimeric constructs relies on a mechanism of reduction of dimensionality [5]: binding of the 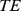 to its receptor restricts the 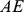 search for its complementary receptor to a small volume close to the cell surface, increasing the chance to interact with it and producing the desired effect. As chimeric ligand-receptor interactions are in many cases limited by receptor diffusion [4], [26], molecular dynamics simulations can be instrumental to properly select the optimal balance between affinity and dissociation rates of 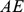 and 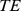 to achieve maximum selectivity, or to determine the length of the protein linker between 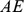 and 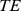.

To test our coarse-grained description of the chimeric ligand-receptor interaction, we implement a chimeric ligand formed by two subunits representing the 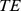 and the 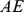 coupled by a linker, (Fig. 4). The linker is usually a polypeptide chain composed of identical subunits [26]. Polypeptide chain dynamics can be well described by a Worm-Like Chain model [27]; therefore the linker is modeled here as an effective force between both subunits given by a Worm-Like Chain interaction [28]:
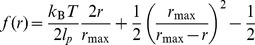
(11)where 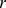 is the distance between 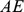 and 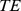, and 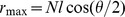 is the size of the polymer chain when completely stretched. 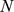 represents the number of monomers in the chain, 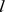 the size of each monomer, and 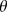 the angle between monomers. The persistence length is given by 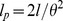.

This force leads to an average end-to-end distance between 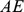 and 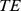 that follows
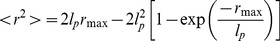
(12)


Two different types of receptors, referred as 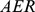 and 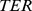, are implemented in our model. They are allowed to interact specifically with the 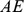 or the 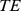 subunit of the chimera via Eqs.2, 3 and 4, while non complementary ligands and receptors interact via a purely repulsive steric interaction:
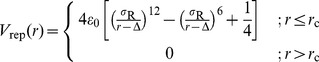
(13)where 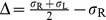 and 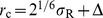. Elements of the same type, i.e., ligand-ligand or receptor-receptor, interact also repulsively through Eq. (1). In this situation, receptor diffusion cannot be neglected since it allows receptors of different type to get close enough for a chimeric ligand to bind to them simultaneously.

**Figure 5:**
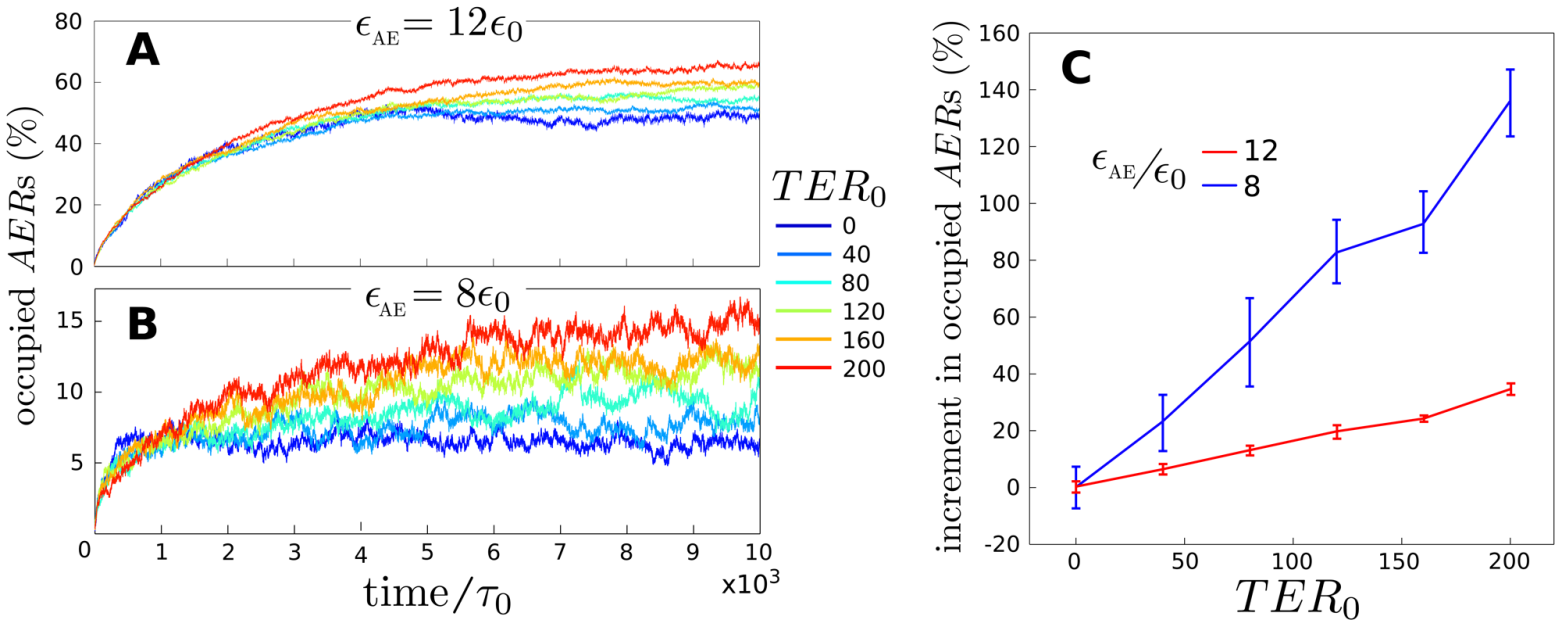
Binding specificity. **A**) and **B**): Percentage of occupied activity receptors as a function of time for different number of total target element receptors (color lines) for (**A**) high (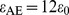) and (**B**) low 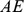 affinity (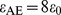). **C**): corresponding proportional increment in the number of activity complexes at equilibrium versus the total number of 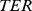
s for the low (blue) and high (red) 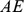 affinities of the previous panels. Simulations are performed for diffusions 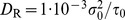, 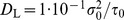; polymer length 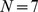; number of receptors 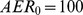, and chimeras at concentration 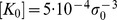. The 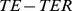 affinity rate is set to 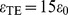, and geometric factors to 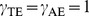. Each trajectory is the result of averaging over 12 independent simulations. Lines connecting points are represented as a guide to the eye.

The selective potential of chimeric ligands against specific cell types relies on the differential expression of the 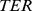 in different cells in a tissue [4], [26]. To study the dependence of the chimeric efficiency on the amount of 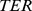 in the cell surface, we compute the number of *AE-AER* complexes formed in chimeric versus monomer configuration for different initial abundances of targeting element receptors, 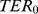. Results are plotted in Fig. 5 for two different situations: high (Fig. 5A) and low (Fig. 5B) interaction strengths of the 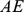 towards its corresponding receptor. As expected, when no 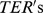 are present, the number of *AE-AER* complexes formed is equivalent to a non-chimeric ligand in both situations (dark blue in Figs. (5A–B). At high affinity rate values of the 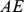, increasing the number of 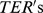 results in a maximum relative increase of 35% in the amount of active complexes (red in Fig. 5C). For reduced values of the 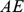 affinity rate, the relative change in activity reaches 140% (blue in Fig. 5C). Increasing the geometric factor 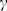 further reduces the affinity rate of the interaction, as shown in Fig. (3). Accordingly, rising the value of 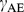 enhances the specificity of the chimeric ligands for both high and low interaction strengths, as shown in Fig. S1 in Supplementary Information. We therefore conclude that the specificity of the chimeric ligands towards cells overexpressing *TER*s is enhanced when the affinity of the *AE*s towards their receptors is reduced. This result is consistent with experimental observations by Cironi and coworkers [26], where chimeras with different mutants of the 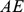 showing reduced affinity exhibit higher selectivity(discrimination of healthy versus unhealthy cells) compared to the monomer.

**Figure 6:**
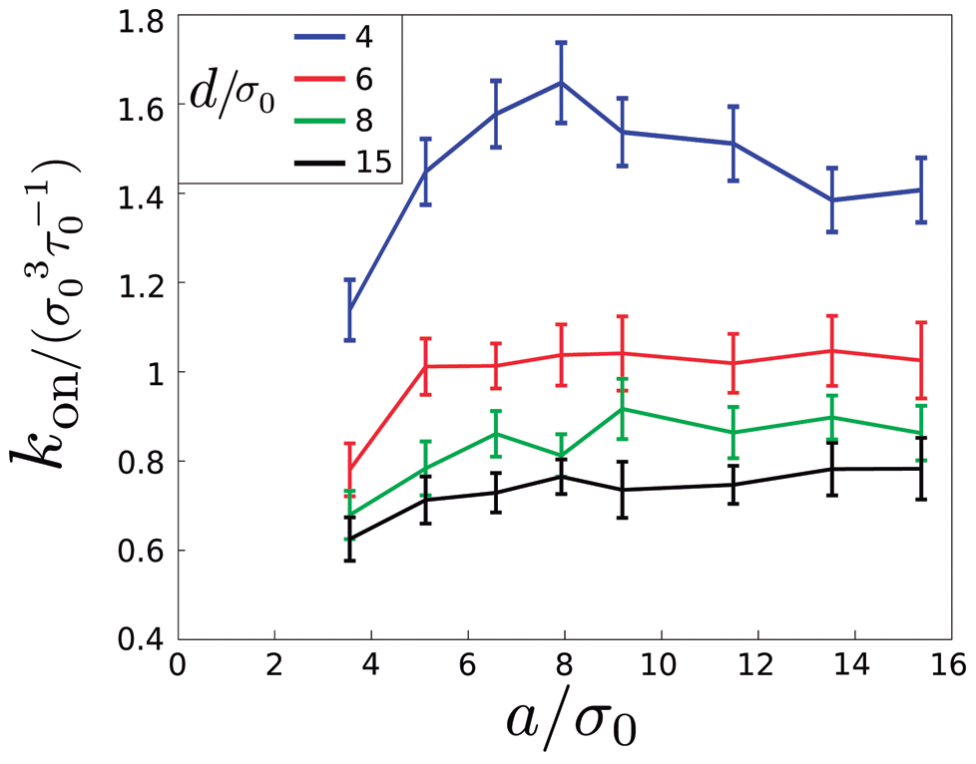
Effect of the linker length. Affinity rate as a function of the linker length for different average distances between receptors. The simulations were done for 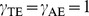, 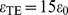, and 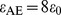. Each outcome is the average of 32 independent simulations. Lines connecting points are represented as a guide to the eye.

Apart from the kinetic rates of both 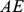 and 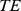 towards their corresponding receptors, a key feature in chimera design is the length of the linker between both subunits. To avoid *in vivo* cleavage of the polypeptide linker chain and other undesired effects, the linker is often engineered with the shortest possible length required for both chimeric subunits to bind simultaneously to both 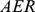 and 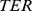 receptors subtypes [26]. In principle, linkers longer than the minimum length could also favor the chance of 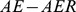 formation by facilitating the encounter of a 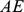 subunit with its receptor after 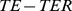 binding, counteracting in this way the slow two-dimensional receptor diffusion. To analyze that, we perform simulations to compute the dependence of the effective affinity rate of 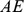
s for different linker lengths and for different average distances between 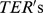 and 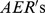 (Fig. 6). We initially set freely diffusing chimeras at a concentration of 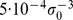 interacting with 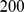 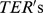 and 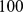 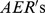. Receptor concentrations are varied by adjusting the size of the x-y plane of the simulation box as indicated in the previous sections, leading to different average distances between target and activity receptors. The vertical size of the simulation box is set to 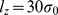, and the interaction parameters are set to 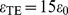, 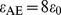, 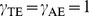.

The linker length 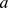, is given by the end-to-end-distance of the worm-like chain approximation described by Eq.12, 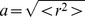. The average distance between receptors, 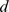, is calculated as follows: for a given number of 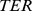
s distributed within a surface 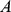, we define an average area per 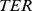 as 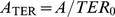, with an associated radius 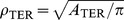. Any 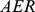 in the surface will be at a distance 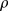 from a 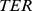 between 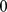 and 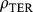. We therefore define 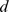 as the average value of 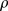:
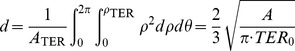
(14)


Results plotted in Fig. 6 show an increase in 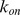 as the distance between receptors decreases, due to the limiting role of receptor diffusion in the dynamics of 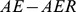 formation. For very short distances (mimicking the situation of receptors clustered in lipid rafts on the cell membrane [15], [29]), receptor diffusion does not limit the binding of the 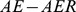 complex, so the effective 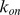 is maximized (blue line). Since, in principle, the mechanism of reduction of dimensionality is more effective for shorter linker lengths (i.e., the 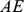 is maintained closer to the membrane after 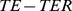 binding), it could be expected that the optimal design would correspond to the shortest linker capable of reaching the two complementary receptors simultaneously (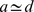). We see, however, that the maximum affinity for each given 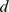 is achieved at lengths of the linker larger that the minimal. For very short receptor distances (blue line), values of the linker around 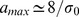 maximize the effective affinity. This is due to effects of linker chain stiffness [30], which we confirmed performing simulations with binding exclusively on the surface, without chimeras in solution. At linker lengths longer than 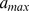, the effect of dimensionality reduction decreases, and so thus the effective affinity rate for clustered receptors. When receptors are not clustered (red, green and black lines), receptor diffusion plays a major role by limiting the interaction [21], so larger linkers facilitate the reaction by counteracting the low receptor diffusion, slightly increasing 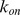 as expected. Overall, the model shows that, in both situations of receptor clustered or far apart, linkers slightly longer that the minimum distance between receptors, (i.e., larger than the one used in [26]), increase the affinity rate of the chimeras, amplifying this way the selectivity of the chimera towards cells overexpressing the targeting element receptor.

## Discussion

In this work we have introduced a tunable coarse grained model for the simulation of ligand-receptor interactions. Our model represents a trade-off between a detailed description of the spatial aspects of the interaction and computational efficiency. Basic spatial features are described by a geometric factor accounting for the directional specificity of the interaction, while ligand-receptor affinity is modulated by the depth of a Lennard-Jones potential. This approach captures relevant spatial constraints while allowing for an explicit description of the diffusive dynamics. The simplicity of the model, together with the computational efficiency of the presented algorithms, facilitates the application of the model to study many-particle systems with geometrical constraints or multiple interactions that cannot be explicitly solved with theoretical considerations of molecular binding or with all-atom simulations. We note, however, that other potentially relevant geometric aspects of the ligand-receptor interaction, such as asymmetry and steric complementarity of the binding pocket and ligand can not be captured by this model.

The model applied to a monovalent ligand-receptor interaction allows to statistically determine the values for the affinity and dissociation rates and link it to the model parameters. The reduced dependence of the dissociation rates on the strength of the ligand-receptor interaction (Fig. 3B) allows to easily find values for 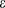 and 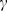 to fit different combinations of 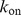 and 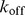. We applied the model to a situation in which typical diffusion time-scales are much larger than binding time-scales, characteristic of a diffusion limited-regime [22]. While, in principle, a reaction-limited regime could also be explored by means of the present model by increasing either 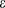 or 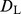, this would result in much longer simulations, as it occurs with many other coarse grained models [31]. We thus focus on applications where geometric, spatial and diffusion effects are important.

As a proof of concept of the capabilities of this type of coarse-grained models applied to ligand-receptor systems, we implemented within this framework a model for chimeric ligands to study the dependence of their selective potential on the concentration of both receptor subtypes, affinity of ligand subunits towards their corresponding receptor, or linker length. Our model shows that the selectivity of the chimera towards cells over-expressing 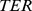 is increased when the intrinsic affinity of the 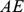 towards 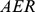 is reduced. This is consistent with the observations reported experimentally by Cironi and coworkers [26], where they used a mutant form of the human interferon 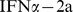 with reduced affinity to improve the selectivity of the treatment towards EGFR-overexpressing cells.

Experimentally, longer linkers are more difficult to implement due to potential cleavage of the linker (basically, due to multiple repeats of the same amino acid sequence in the linker), so the shortest linker possible is often implemented experimentally [26]. Analysis of the effect of the linker length shows that linker lengths longer than the minimum distance between receptors increase the effective affinity of the 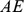, in both situations of clustered and non clustered receptors. In the case of clustered receptors, the model predicts an optimal linker length that depends on structural properties of the linker itself, such as the stiffness of the amino acid chain.

The presented coarse grained model represents a powerful tool to understand general properties of complex ligand-receptor systems with no analytical solutions available, or where analytical approximations remain to be validated. This is the case of systems with many agents involved, multi-interactions between species or situations with special geometries. Some examples could involve receptor dimerization and its influence on binding, multivalent binding and receptor cross-linking. A careful definition of the geometric factor would also allow multiple ligands to bind simultaneously to the same receptor. In addition, the coarse-grained approximation could be implemented as a module for more complex systems including downstream signaling propagation, or even cell membrane dynamics described via generic coarse grained models, opening the possibility to study the effects of membrane fluctuations or membrane inhomogeneties on ligand-receptor dynamics.

## Supporting Information

Figure S1
**Binding specificity as a function of** 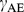 **.** Proportional increment in the number of activity complexes at equilibrium versus the total number of 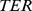
s for different 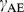 (see figure legends), given high (**A**) and low (**B**) 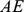 interaction strengths. Simulations are performed for diffusions 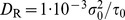, 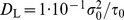; polymer length 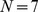; number of receptors 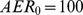, and chimeras at concentration 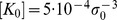. The 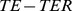 affinity rate is set to 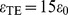, and the geometric factor to 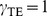. Each trajectory is the result of averaging over 12 independent simulations. Lines connecting points are represented as a guide to the eye.(TIF)

Software S1 **Collection of source code files to compile and run the coarse-grained simulations used in this manuscript can be found in the Software S1 supplementary file.**
(BZ2)
