# Supplementary figures and images for: A Tunable Coarse-Grained Model for Ligand-Receptor Interaction
Source: PLoS Comput Biol. 2013 Nov 14;9(11):e1003274. doi: 10.1371/journal.pcbi.1003274 (PMC3828130; doi:10.1371/journal.pcbi.1003274)

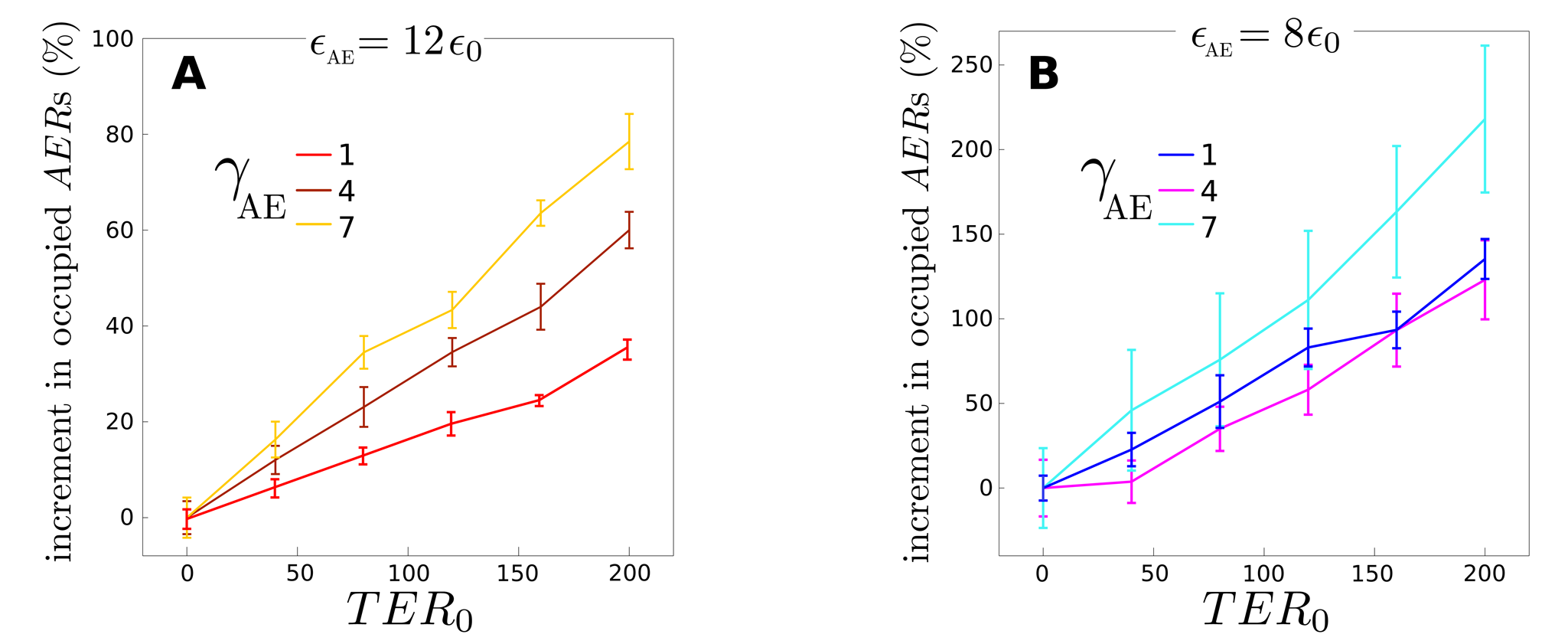

Supplement: Figure S1 — Binding specificity as a function of . Proportional increment in the number of activity complexes at equilibrium versus the total number of s for different (see figure legends), given high (A) and low (B) interaction strengths. Simulations are performed for diffusions , ; polymer length ; number of receptors , and chimeras at concentration . The affinity rate is set to , and the geometric factor to . Each trajectory is the result of averaging over 12 independent simulations. Lines connecting points are represented as a guide to the eye. (TIF) [file pcbi.1003274.s001.tif]
